# Comparative Evaluation of Bleaching Efficacy on White Spot Lesions Managed With Resin Infiltration and Remineralization Using Casein Phosphopeptide-Amorphous Calcium Phosphate: An In Vitro Dynamic pH Cycling Study

**DOI:** 10.7759/cureus.113876

**Published:** 2026-08-03

**Authors:** Rishika Raman, Somya Kumari, Chi Koy Wang, Abhinav K Singh, Sanmitra Saha

**Affiliations:** 1 Department of Conservative Dentistry and Endodontics, Buddha Institute of Dental Sciences and Hospital, Patna, IND

**Keywords:** casein phosphopeptide-amorphous calcium phosphate, dental bleaching, resin infiltration, tooth remineralization, white spot lesion

## Abstract

Background: Bleaching is often desired by patients, but with white spot lesions (WSLs), this procedure can be a cause of concern due to its action on demineralized enamel. Treating these WSLs before bleaching can be a treatment modality to alleviate the action of the bleaching agent. This study assessed the efficacy of a 40% hydrogen peroxide bleaching agent on demineralized enamel pre-treated with resin infiltration (RI) or casein phosphopeptide-amorphous calcium phosphate (CPP-ACP) using a dynamic pH cycling model to simulate oral conditions.

Materials and methods: Fifty-six human molar enamel samples were randomly divided into four groups after staining with coffee solution, based on the surface treatment before bleaching (n=14): Group I, sound enamel/control and no treatment (NT); Group II, untreated artificial caries (AC) and NT; Group III, AC and was treated with RI ICON® (DMG Chemisch-Pharmazeutische Fabrik GmbH, Hamburg, Germany); and Group IV had AC and was remineralized (CPP-ACP, GC Tooth Mousse; GC Corporation, Tokyo, Japan). AC was induced via dynamic pH cycling (12 hours demineralization solution/12 hours artificial saliva) over seven days. Groups III and IV were treated for seven days. All groups were then bleached using 40% hydrogen peroxide (Opalescence Boost™; Ultradent Products, Inc., South Jordan, UT, USA) for 40 minutes. Color changes (ΔE*) were measured using a spectrophotometer, and surface topography was analyzed using scanning electron microscopy (SEM) prior to bleaching and 14 days after bleaching. A one-way ANOVA and Tukey’s post-hoc test for intergroup comparison were done.

Results: All groups demonstrated color change beyond the clinical visibility threshold (ΔE*>3.3). Group IV demonstrated the least color change (5.18, p < 0.05). The bleaching efficacy in Group III was not significantly different from Group I. Group II exhibited the highest mean color change. The changes in enamel surface seen the least in surface treated groups were minimal compared to the untreated demineralized group (Group II). SEM analysis revealed that while bleaching caused significant surface irregularities and prismatic exposure in the untreated demineralized group (Group II), RI and CPP-ACP pre-treatments mitigated this damage, with CPP-ACP samples exhibiting the smoothest post-bleaching surface topography.

Conclusion: RI is the most effective treatment modality for enhancing esthetic outcomes, while CPP-ACP provides better protection to the enamel damaged by the bleaching agent, although the color change after bleaching is less than in a window of a seven-day treatment period in this study.

## Introduction

White spot lesions (WSLs) are the earliest stage of demineralization on the enamel surface that can be perceived by the human eye. These lesions can be very commonly seen in patients undergoing orthodontic treatment. Lack of proper oral hygiene also leads to discoloration, and after the removal of fixed appliances, some WSLs persist [1]. To enhance their smile post-orthodontic treatment, it is common for patients to undergo bleaching.

Bleaching agents like hydrogen peroxide or carbamide peroxide seep through the enamel. They break the weak bonds between the larger stain molecules and the organic matrix into smaller, less complex molecules [2]. Due to the breakdown of complex molecules into smaller molecules, there is a drop in pH of the bleaching agent, which results in loss of mineral content in the enamel [3]. Loss of mineral content leads to increased porosity and, consequently, demineralization. At this stage, treatment of demineralization can stop the progress of the lesion, and thus, dental caries can be avoided [4].

Remineralization can be attempted to treat the subsurface demineralized lesion. Casein phosphopeptide-amorphous calcium phosphate (CPP-ACP) is one of the methods via which remineralization can be done. It stabilizes calcium and phosphate ions in a soluble, amorphous state that is capable of releasing these ions when the pH falls. These ions diffuse into the enamel rods, where they form hydroxyapatite crystals, repairing WSLs [5-8].

Resin infiltration (RI) is an ultraconservative approach to arrest initial carious lesions, whereby the low-viscosity resin is used to occlude the highly porous surface of incipient enamel lesions [9]. This material constructs a three-dimensional covalent polymer network that helps restore lost mineral content. By microscopically interlocking with the remaining enamel prisms, it creates a physical shield against hydrogen ions. This barrier effectively prevents further acid-induced demineralization and halts the progression of subsurface lesions [9, 10]. ICON® infiltrant (DMG Chemisch-Pharmazeutische Fabrik GmbH, Hamburg, Germany) has a low viscosity, low contact angle, high surface tension, and high penetration effect coefficient with a refractive index of 1.48, which is close to that of healthy enamel (refractive index 1.65), and thus the WSLs appear similar to the surrounding sound enamel, creating a chameleon effect [11, 12]. However, RI does not whiten the tooth surface, as there is no change in the shade of the tooth. To combat this, the synergistic effect of RI and bleaching has been suggested to enhance esthetic outcomes [13]. 

One of the previous studies investigated the effect of bleaching on WSLs treated with RI and CPP-ACP over a period of 14 days and concluded that bleaching after RI achieved a color change that was similar to that of sound enamel. However, this study was limited to a static pH demineralization cycle to create WSLs, which is not how the oral cavity behaves [14].

In this study, the WSLs are formed with dynamic cycling of pH, where a period of demineralization and remineralization occurs in tandem. The purpose of this study is to analyze the surface morphology of enamel pre- and post-bleaching after treatment with RI and CPP-ACP using scanning electron microscopy (SEM) as well as the effectiveness of bleaching on the color after treatment with RI and CPP-ACP using a spectrophotometer.

The objectives of this study are to evaluate and compare the effect of different pretreatments (ICON® RI and CPP-ACP) on bleaching efficacy against sound enamel and demineralized enamel in a dynamic pH cyclic model using spectrophotometry as the primary objective. The effects of bleaching on enamel surface morphology are also analyzed using SEM as the secondary objective.

The first null hypothesis to be tested is that pre-treatment of demineralized enamel with ICON® RI or CPP-ACP for seven days followed by 40% hydrogen peroxide bleaching would not result in a significant difference in bleaching efficacy compared to control groups, as assessed by spectrophotometry. The second null hypothesis is that bleaching with 40% hydrogen peroxide would not induce significant surface morphological changes on enamel pretreated with RI or CPP-ACP, as assessed by SEM.

## Materials and methods

Study design

**Figure 1:**
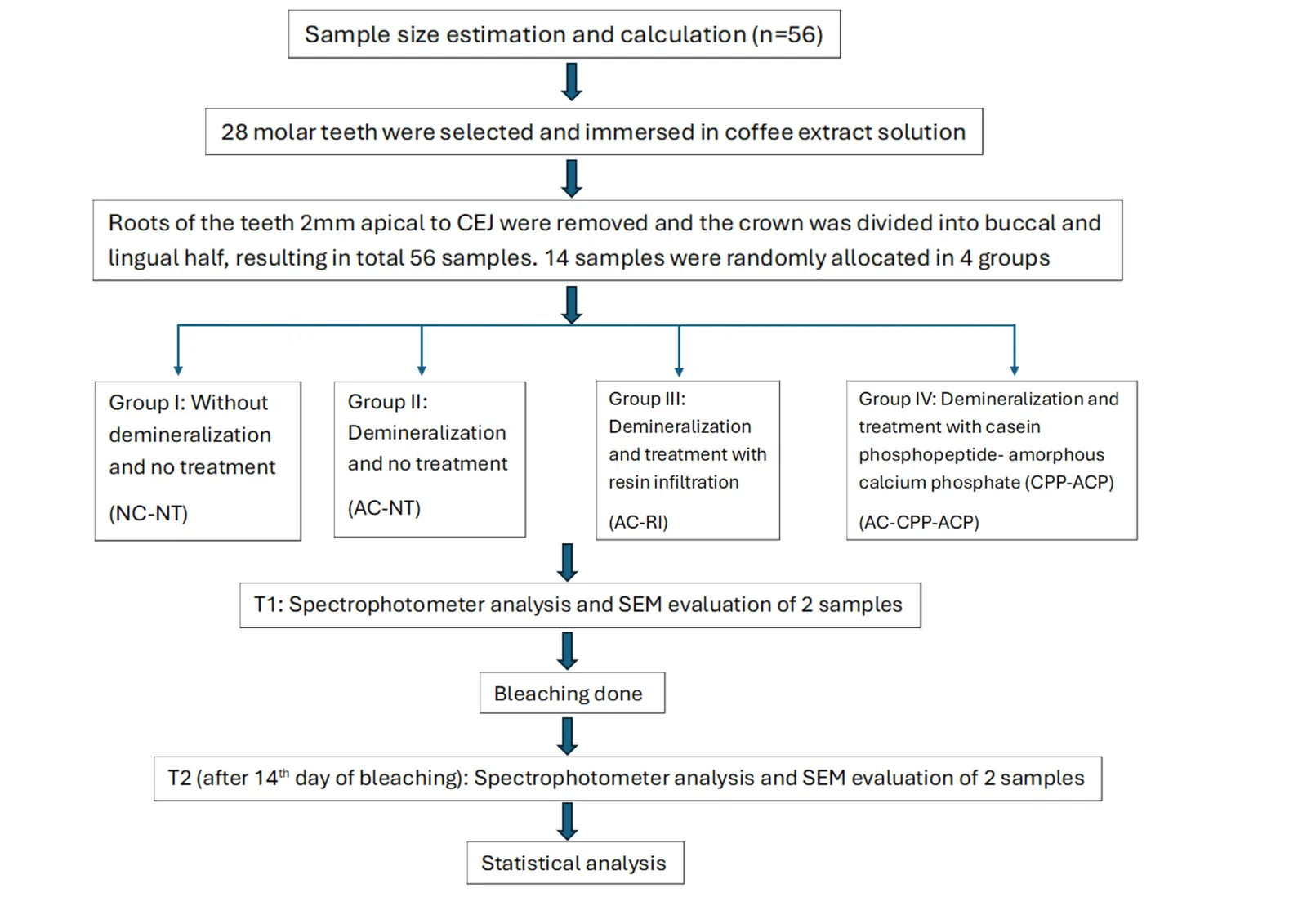
Study flow diagram CEJ: cementoenamel junction; NC: without demineralization/no artificial caries; NT: no treatment; AC: artificial caries; RI: resin infiltration; SEM: scanning electron microscopy

An in vitro study was designed to assess the efficacy of bleaching on demineralized enamel treated with RI and CPP-ACP. The study protocol was approved by the Institutional Ethical Committee, Buddha Institute of Dental Sciences & Hospital, Patna, Bihar, India (Ref. No. 78/BIDSH/IEC/EXEMP/2024(2023-2024) prior to the collection of extracted human molars, ensuring compliance with the ethical guidelines for the use of human biological materials. The flow diagram provides an overview of the study as shown in Figure 1.

Sample selection and storage

Sample size was calculated using G*Power software (Version 3.1.9.4; Heinrich Heine University Düsseldorf, Düsseldorf, Germany), keeping a Type I error of 5% and 90% of power (1-β) with an effect size of 0.524, according to a study done by Jacob et al. [14]. This gave a total estimate of 56 samples, i.e., n=14 in each of the four groups. 28 non-carious human permanent molars were extracted and stored in artificial saliva (Torion Lifesciences, Jaipur, Rajasthan), which contained carboxymethylcellulose (1% w/v), sorbitol (3% w/v), potassium chloride (0.12% w/v), sodium chloride (0.0844% w/v), magnesium chloride (0.0052% w/v), calcium chloride (0.0146% w/v), and potassium dihydrogen phosphate (0.0342% w/v) at room temperature until testing, which was renewed daily. Teeth with enamel defects, stains, visible cracks, or restorations were excluded from the study.

Staining procedure

Thorough scaling was done to remove any calculus or debris present. The teeth were artificially stained using a coffee extract solution by dissolving two packets of 3.6 gm instant coffee powder (Nescafé Classic, Nestlé S.A., Vevey, Switzerland) in 100 mL of distilled water at 100°C. The teeth were soaked in coffee solution overnight and ultrasonicated to obtain a shade of A3 or darker according to the VITA Classical Shade Guide (VITA Zahnfabrik, Bad Säckingen, Germany), arranged in a value-based format.

Sample preparation

The roots of the teeth 2mm apical to the cemento-enamel junction (CEJ) were removed using a diamond disk (NMD Nexus Medodent, Mumbai, India), leaving the coronal portion of the teeth. They were mesiodistally sectioned into two halves: buccal enamel and lingual enamel surfaces, resulting in a total of 56 samples, which were independently allocated using a computer-generated random number table. They were sequentially polished using silicon carbide paper grits to obtain an area of 10mm in diameter and 2 mm in thickness.

The samples were randomly allocated into four groups (n=14): Group I, without demineralization/no artificial caries (AC) and no treatment (NT); Group II, demineralization without any treatment (AC-NT); Group III, demineralization and treatment with RI (AC-RI); Group IV, demineralization and treatment with CPP-ACP (AC-CPP-ACP)

Demineralization procedure

Forty-two enamel samples (Groups II-IV) where AC, i.e., demineralization, was to be induced, were immersed in a demineralization solution for 12 hours, alternating with 12 hours of artificial saliva to mimic the oral cavity conditions for seven days. The demineralization solution contained 2.2 mM potassium dihydrogen phosphate, 2.2 mM calcium chloride, 0.050 M acetic acid, and 1 M potassium hydroxide, making the pH 4.6. The samples were then rinsed and stored in distilled water.

Resin infiltration

After demineralization, 14 samples, which were allocated to Group III (AC-RI), were treated with ICON® RI (DMG Chemisch-Pharmazeutische Fabrik GmbH, Hamburg, Germany). The ICON® Etch syringe, containing 15% hydrochloric acid gel, was used after activating it for two minutes. ICON® Etch was aspirated, and the enamel samples were rinsed with water for 30 seconds. The samples were dried using oil-free and water-free air. ICON® Dry, containing 99% ethanol, was applied for 30 seconds and then air-dried. An ICON® infiltrant, containing low-viscosity resin, was applied and left on the enamel for three minutes. Excess infiltrant was removed, light-cured for 40 seconds, and polished with discs (Super Snap Mini-kit, Shofu Inc., Kyoto, Japan).

CPP-ACP remineralization

After demineralization, 14 samples were allocated to Group IV (AC-CPP-ACP); 0.1 mL of CPP-ACP (GC Tooth Mousse; GC Corporation, Tokyo, Japan) was applied on enamel surfaces with the help of an applicator tip and left for a minimum of three minutes. Samples were stored in artificial saliva, which was renewed after 12 hours. This step was repeated five times a day over a week.

Bleaching procedure

The bleaching agent (Ultradent Opalescence Boost™, South Jordan, Utah, USA), with a whitening gel composition of 40% hydrogen peroxide chemically activated gel, was freshly prepared, and a thickness of 0.5-1 mm was applied to the tooth surface and left for 20 minutes for one cycle. The gel was wiped, and the bleaching cycle was repeated once more, resulting in a total bleaching time of 40 minutes as per the manufacturer’s instructions. The samples were rinsed with distilled water, blot-dried, and stored in a salivary substitute for two weeks, which was renewed daily.

Color shade assessment

The spectrophotometer shade assessment was done in two stages using a calibrated spectrophotometer (Konica Minolta CM 5, Konica Minolta Inc., Tokyo, Japan) under standardized D65 illumination with Spectramagic NX software on all the samples from Group I to Group IV to measure Commission Internationale de l'Éclairage (CIE) L*a*b* values. A custom silicone positioning jig with a central window was used to standardize the area to be evaluated, and three measurements were used to calculate a mean value.

T1 (baseline/ pre-bleach): After treatment with RI and CPP-ACP, all the samples from Group I-IV were assessed; T2 (post-bleach): After 14 days from the bleaching procedure, all the samples from Group I-IV were assessed.

The three coordinate values (L* a* b*) of the CIE were used to determine the color of an object in a three-dimensional space, where the L* axis represented the degree of lightness of the sample with values ranging from 0 (black) to 100 (white). The degree of green/red color was represented by the a* plane, whereas the b* plane represented the blue/yellow color plane. The same operator performed all measurements to ensure consistency throughout the study, and the data was tabulated into a Microsoft Excel sheet (Microsoft Corp., Redmond, WA, USA). The following formula was used to determine the color change (ΔE*) using the CIE 1976 formula:

$&Delta;E^\ast=\left(\sqrt{{\left({\varDelta L}^{\ast }\right)}^{2}+{\left({\varDelta a}^{\ast }\right)}^{2}+{\left({\varDelta b}^{\ast }\right)}^{2}}\right)\displaystyle \text{}$, where Δ represents the difference between the post-bleaching values and baseline values. 

SEM assessment

Enamel surface topography was analyzed on two dedicated extra samples from each group before and after 14 days of bleaching via scanning electron microscope (ZEISS Gemini SEM 500, Carl Zeiss Microscopy GmbH, Jena, Germany). The samples were stored in ethyl alcohol for eight hours and left to dry overnight. After mounting and gold sputtering, SEM was performed at 5.00kV at 1000x magnification on all samples. Two samples were tested at baseline, and two different samples were tested after 14 days of bleaching. The best representative image was selected, which had a low signal-to-noise ratio and no artifacts were noted.

Statistical analysis

The data were analyzed using IBM SPSS Statistics software, version 21.0 (IBM Corp., Armonk, NY, USA). Normality was confirmed using the Shapiro-Wilk test. To evaluate the changes within each study group from baseline (Pre) to the final assessment (Post), a paired t-test was employed. For comparisons between the four study groups, a one-way Analysis of Variance (ANOVA) was performed. When the ANOVA indicated a statistically significant difference (p < 0.05), Tukey’s Honestly Significant Difference (HSD) post hoc test was applied to identify specific pairwise differences between groups. A p-value of less than 0.05 (p < 0.05) was considered statistically significant.

## Results

Color changes

The results of color assessment pre- and post-bleaching were subjected to statistical analysis to evaluate the bleaching efficacy of 40% hydrogen peroxide following WSL treatment. 

For the color co-ordinate a*, Group II and Group III demonstrated statistically significant changes, with p-values of 0.028 and 0.036, respectively (p < 0.05), as shown in Table 1. In contrast, the changes observed in Group I (p = 0.125) and Group IV (p = 0.806) did not reach statistical significance. The statistical analysis revealed that baseline values were comparable across all four groups with no significant differences observed (F = 0.238, p = 0.869). Similarly, no significant differences were found between the groups following the intervention (F = 1.272, p = 0.294).

**Table 1:** Comparison of pre- and post-treatment of a* color co-ordinate values within and between experimental groups Data are presented as mean and standard deviation (SD). Intra-group comparisons between baseline (pre-treatment) and post-treatment intervals were evaluated using the paired Student's t-test. (a*) denotes the red-green axis Inter-group comparisons across all four experimental groups were evaluated using ANOVA, revealing no statistically significant differences at baseline (F = 0.238, p = 0.869) or post bleaching (F = 1.272, p = 0.294). (*) Indicates a statistically significant difference within the group (p < 0.05); F: ANOVA F statistic

Groups	Pre a* mean	Pre a* SD	Post a* mean	Post a* SD	t value	p-value
Group I	1.14	1.21	0.31	1.85	1.640	0.125
Group II	1.13	1.32	-0.31	1.57	2.469	0.028*
Group III	0.90	1.05	-0.35	1.78	2.342	0.036*
Group IV	0.82	1.31	0.69	1.43	0.251	0.806

All four experimental groups showed a statistically significant reduction in mean scores from the "pre b*" to "post b*" phases, as evidenced by p-values ranging from 0.004 to <0.001 in Table 2. Group II demonstrated the most substantial decrease, dropping from a mean of 7.25 to 2.17. A comparison between the groups at the baseline (Pre b*) revealed a significant difference (p = 0.02), primarily driven by Group I's higher starting mean (8.85) compared to the significantly lower means of Groups III and IV (7.11). However, by the post b* phase, the differences between the four groups were no longer statistically significant (p = 0.07), suggesting that the intervention served to equalize the outcomes across all samples regardless of their starting points.

**Table 2:** Comparison of pre- and post-treatment b* color co-ordinate values within and between the experimental groups. Data are presented as mean and standard deviation (SD). (b*) denotes the yellow-blue axis. Intra-group comparisons between baseline (pre) and post-treatment intervals were evaluated using the paired Student's t-test. Inter-group comparisons across all four experimental groups were evaluated using One-way ANOVA, revealing a statistically significant difference at baseline (F = 3.562, p = 0.02) but no statistically significant difference post-bleaching (F = 2.493, p = 0.07). Same superscript lowercase letters within the same vertical column indicate homogeneous subsets that are not statistically significantly different from one another based on Tukey's post-hoc test (p > 0.05) (*) indicates a statistically significant difference (p < 0.05); F: ANOVA F statistic

Groups	Pre b* mean	Pre b* SD	Post b* mean	Post b* SD	t value	p-value
Group I	8.85^a^	1.39	5.72	2.73	4.365	0.001*
Group II	7.25^ab^	1.81	2.17	4.18	5.903	<0.001*
Group III	7.11^b^	1.85	4.11	4.16	3.427	0.004*
Group IV	7.11^b^	1.66	4.44	2.45	3.784	0.002*

The comparative analysis of pre-L* and post-L* values across the four study groups revealed varying levels of statistical significance in Table 3. Within-group comparisons showed that Group III experienced a statistically significant increase from pre-L* (Mean = 71.82) to Post-L* (Mean = 74.19), with a p-value of 0.012. In contrast, although Groups I and II showed numerical increases in mean values, these changes only approached marginal significance (p = 0.052 and p = 0.058, respectively). Group IV showed the least change, which was not statistically significant (p = 0.243). Furthermore, inter-group comparisons indicated no statistically significant differences between the four groups at either the pre-L* stage (p = 0.056) or the post-L* stage (p = 0.327), suggesting baseline homogeneity and a uniform distribution of outcomes across the groups following the intervention. There were no significant differences between the groups during the post-hoc analysis.

**Table 3:** Comparison of pre- and post bleaching L* color co-ordinate values within and between the experimental groups. Data are presented as mean and standard deviation (SD). (L*) depicts lightness Intra-group comparisons between baseline (pre) and post-treatment intervals were evaluated using the paired Student's t-test. Inter-group comparisons across all four experimental groups were evaluated using One-way ANOVA, revealing no statistically significant differences at baseline (F = 2.686, p = 0.056) or post bleaching (F = 1.178, p = 0.327); hence, Tukey's post-hoc test was not done. (*) indicates statistically significant difference (p < 0.05); F: ANOVA F-statistic

Groups	Pre L* mean	Pre L* SD	Post L* mean	Post L* SD	t value	p-value
Group I	73.70	3.22	76.95	6.41	-2.319	0.052
Group II	70.63	2.98	73.76	4.57	-2.074	0.058
Group III	71.82	3.14	74.19	3.35	-2.940	0.012*
Group IV	73.47	3.83	74.64	4.76	-1.222	0.243

The mean changes in Δa*, Δb* and ΔL* and the total change ΔE* for all study groups are summarized in Table 4. Regarding the individual co-ordinates, no statistically significant differences were observed among the four groups for Δa* (p = 0.339), Δb* (p = 0.147), or ΔL* (p = 0.621), indicating a relatively uniform shift. However, a statistically significant difference was identified in the total change ΔE* among the groups (p = 0.015). Pairwise comparisons using Tukey’s post hoc test revealed that Group II exhibited the highest mean change (8.61), which was significantly greater than that of Group IV (5.18, p < 0.05). While Group I (6.77) and Group III (5.86) showed intermediate values, they did not differ significantly from either Group II or Group IV (p > 0.05). These results suggest that the experimental conditions in Group II led to the most pronounced overall color change compared to the other groups.

**Table 4:** Comparison of color change parameters between the experimental groups Data are presented as mean and standard deviation (SD). Inter-group comparisons across all four experimental groups were evaluated using one-way ANOVA, with the corresponding test statistic (F value) and p-value reported in dedicated columns. Same superscript lowercase letters (^a^, ^b^, ^ab^) within the same horizontal row indicate homogeneous subsets that are not statistically significantly different from one another based on Tukey's post-hoc test (p > 0.05). The absence of superscript letters in the Δa*, Δb*, and ΔL* rows reflects a non-significant overall ANOVA (p > 0.05), meaning post-hoc pairwise comparisons were not applicable. (*) Indicates a statistically significant difference between the groups (p < 0.05); Δa*: change in red-green chromaticity co-ordinate; Δb*: change in yellow-blue chromaticity co-ordinate; ΔL*: change in lightness; ΔE*: total color change

Parameters	Group I mean	Group I SD	Group II mean	Group II SD	Group III mean	Group III SD	Group IV mean	Group IV SD	F value	p-value
Δa*	-0.83	1.90	-1.44	2.18	-1.25	1.99	-0.13	1.99	1.147	0.339
Δb*	-3.14	2.69	-5.08	3.22	-3.01	3.28	-2.67	2.64	1.867	0.147
ΔL*	3.25	5.69	3.13	5.65	2.37	3.01	1.17	3.58	0.595	0.621
ΔE*	6.77^ab^	4.06	8.61^a^	2.78	5.86^ab^	2.07	5.18^b^	2.01	3.796	0.015*

Enamel morphology changes

**Figure 2:**
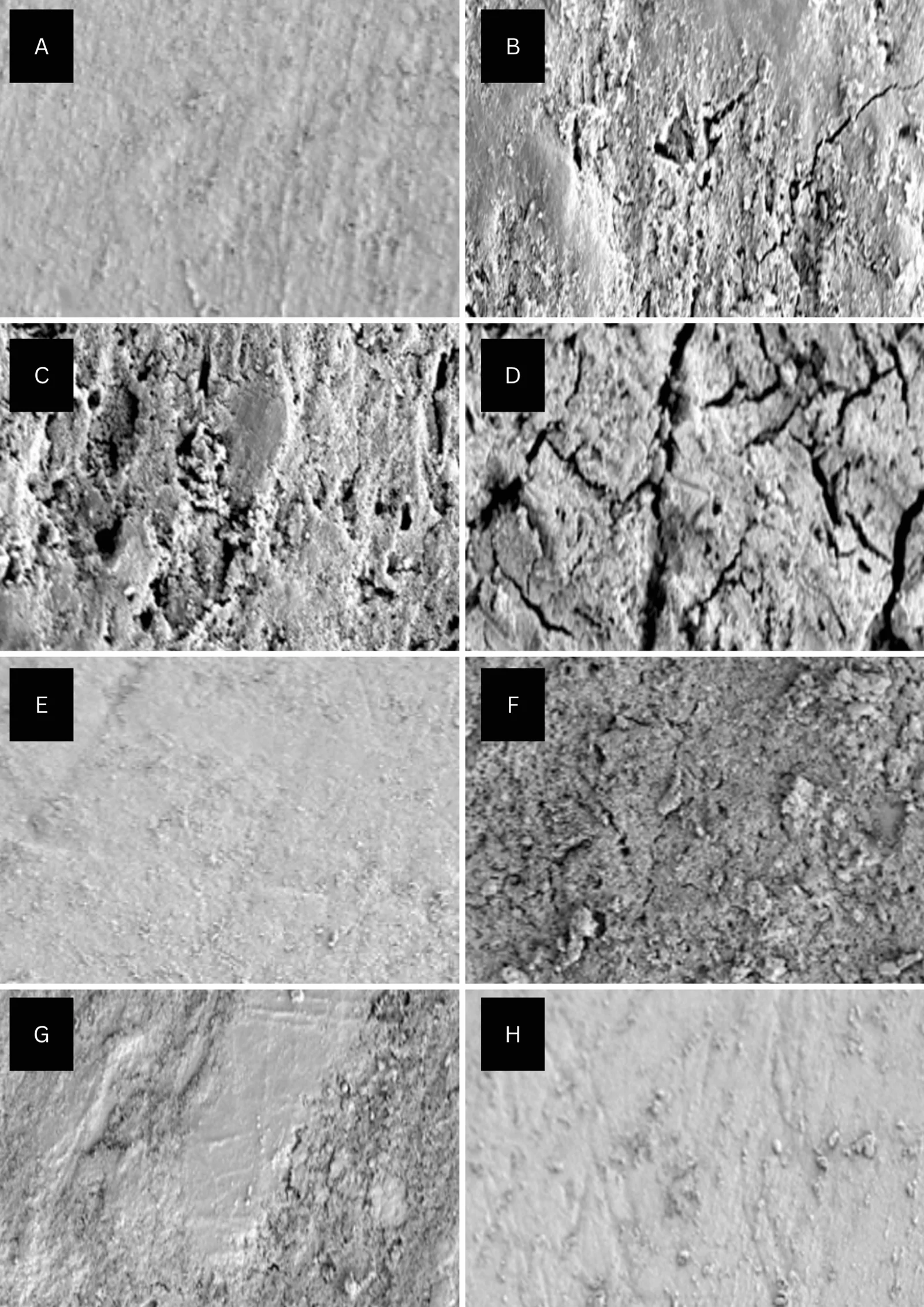
Comparative scanning electron microscopy (SEM) microphotographs of enamel surface morphology at 1000× magnification. (A) Group I (NC-NT) pre-bleaching, displaying intact, smooth enamel prisms with defined rod boundaries. (B) Group I post bleaching, showcasing minor superficial etching and slight prism boundary degradation. (C) Group II (AC-NT) pre-bleaching, depicting artificial subsurface carious structural loss and micro-porosities. (D) Group II post bleaching, demonstrating severe structural collapse, deeper micro-cracks, and erosion patterns. (E) Group III (AC-RI) pre-bleaching, exhibiting an integrated, smooth polymer layer occluding the enamel framework. (F) Group III post bleaching, showing irregular polymer matrix degradation and moderate exposure of underlying prisms. (G) Group IV (AC-CPP-ACP) pre-bleaching, showing a highly homogeneous surface covered by protective mineral precipitation. (H) Group IV post bleaching, revealing diffuse, fine granular micro-deposits distributed across a largely intact surface layer. NC: no artificial caries; NT: no treatment; AC: artificial caries; RI: resin infiltration; CPP-ACP: casein phosphopeptide-amorphous calcium phosphate

Qualitative evaluation via SEM at 1000× magnification of the enamel surface confirmed varying degrees of morphological topography alteration across all groups post bleaching, as shown in Figure 2. Group I initially showed healthy, well-organized hydroxyapatite prisms (Figure 2A), which experienced localized, mild surface irregularities following peroxide exposure (Figure 2B). The demineralized control group (Group II) presented with structural porosity features from AC development (Figure 2C); following bleaching, this architecture suffered advanced morphological breakdown, exhibiting deep surface micro-fissures and severe erosion patterns (Figure 2D).

In the RI group (Group III), the pre-bleaching samples confirmed that the low-viscosity resin successfully filled and masked the initial porous structure, but a slight honeycomb pattern was still visible (Figure 2E). Post-bleaching micrographs revealed irregular variations across the polymer surface layer, pointing to moderate degradation of the resin framework by the aggressive bleaching agent (Figure 2F). Conversely, the application of CPP-ACP (Group IV) yielded a smoothed, well-mineralized external structure prior to bleaching (Figure 2G). Following exposure to 40% hydrogen peroxide, the surface retained structural continuity but showcased widespread, fine granular mineral micro-precipitates scattered across the enamel surface (Figure 2H).

## Discussion

In the present study, all groups demonstrated color change and enamel morphology change due to the intervention of a 40% hydrogen peroxide bleaching agent for 40 minutes, regardless of surface treatment. The color change in all the groups was greater than 3.3 [15], which is the threshold value for clinical color change observation to be detected by the human eye.

For Group I, the primary driver of the bleaching effect was the significant reduction in yellowness (b*), which dropped from 8.85 to 5.72 (p = 0.001). While lightness increased numerically, the change only approached marginal significance (p = 0.052), suggesting that professional bleaching in sound enamel primarily acts through chromatic reduction rather than pure value increase. The qualitative SEM study for the surface topography of Group I revealed unbleached enamel in artificial saliva with slight irregularities on the surface before bleaching, whereas after bleaching, there were increased surface porosities and wide interprismatic areas, which suggests that bleaching of intact enamel may induce localized surface alterations. The oxidative processes initiated by hydrogen peroxide generate free radicals that degrade organic matrices and can also interact with inorganic components, leading to gradual dissolution of the enamel surface and removal of mineral elements [16].

Group II demonstrated the most color instability, with statistically significant reduction in both a* (p = 0.028) and b* (p = 0.0001). Specifically, b* values dropped from 7.25 to 2.17, representing the largest decrease in yellowness across all groups. This highlights that untreated porous lesions are highly susceptible to drastic color shifts as the peroxide diffuses non-selectively through the demineralized matrix. The overall color change was highest in this group, suggesting that the carious lesion body acts as a reservoir for the bleaching gel, allowing for an exaggerated oxidation response. Under the SEM, before bleaching, Group II, which mimicked WSLs with no treatment, displayed slight irregularities of the enamel surface. The damage was exaggerated when the bleaching agent was applied, leading to deep surface porosities and dissolution of the interprismatic areas. This finding is consistent with the studies done by Owda et al. [17] and Jacob et al. [14], where bleached enamel demonstrated extensive damage on the surface.

In Group III, there were statistically significant improvements across all three dimensions of color, i.e., L*, a*, and b*. Notably, it was the only group to achieve a significant increase in lightness, i.e., L* rising from 71.82 to 74.19 (p = 0.012). This leads to the conclusion that the resin-enamel complex is highly responsive to bleaching, allowing for a comprehensive esthetic restoration leading to increased value as well as reduced chroma. The incorporation of RI prior to bleaching thus appears to mitigate the detrimental effects observed in untreated lesions, offering both structural integrity and enhanced esthetic outcomes [16]. This group demonstrated an intermediate degree of overall color change with no statistically significant difference detected compared to the control group (Group I). This finding supports the proposed mechanism that the resin infiltrant behaves as a semipermeable barrier; it is dense enough to arrest the progression of caries and improve mechanical properties, yet porous enough at the molecular level to allow for the passage of oxygen free radicals [13, 18-19]. SEM study of Group III revealed a smooth surface where the underlying etched prisms are partially masked, according to a study done by Arnold et al. [20]. The resin penetrating the enamel can be seen as patches. Bleaching caused moderate damage to the surface, as the resin is supposedly believed to act as a semipermeable barrier, thus allowing the peroxides to penetrate the enamel. However, this mechanistic pathway represents an interpretation rather than a directly measured parameter.

Group IV appeared to be the most resistant to bleaching effects. While this group showed a significant reduction in yellowness (b*, p = 0.002), it showed no significant change in lightness (p = 0.243) or in the red-green axis (a*, p = 0.806). This result supports the reasoning that the remineralized surface acts as a barrier that prevents the bleaching agent from showing its full effect when it comes to color changes. The seven-day remineralization protocol created a dense, mineralized surface layer of calcium and phosphate, and thus, the efficacy of color change due to the bleaching agent was reduced. SEM images of Group IV after bleaching showed a smoother enamel surface than any other group, with a deposit of granular structures, presumably the calcium and phosphate ions that are released by the CPP-ACP complex. The granular layer acts as a physical barrier that fills in the structural defects caused by enamel caries, as was observed in the study done by de Vasconcelos et al. [21]. This is consistent with the SEM images of this study.

The overall color change after bleaching was seen the least in Group IV (CPP-ACP treated) and most in Group II (untreated demineralized). Based on these spectrophotometric outcomes, the first null hypothesis was rejected for the CPP-ACP-treated samples due to their significantly restricted bleaching efficacy, whereas no statistically significant difference was detected between the RI group and sound enamel. Furthermore, because qualitative SEM analysis suggested trends toward distinct micro-morphological alterations and topographical changes across the limited representative samples from all four experimental groups following peroxide exposure, the second null hypothesis was rejected. These morphological observations should be interpreted with caution given the qualitative nature and small sample size of the SEM analysis, and clinical extrapolations should be limited strictly to these in vitro conditions.

Limitations

Since this is an in vitro study, the exact complex oral environment cannot be simulated. The 56 samples were harvested from 28 teeth (buccal and lingual surfaces), which introduced potential non-independence between paired samples that was not controlled for in the statistical model. The staining protocol and demineralization procedure are not reflective of the actual biochemical processes. The use of flat enamel surfaces does not factor in the natural curvature and anatomical variations of the tooth. Remineralization was limited to seven days, while clinically, it might take several weeks or months for the minerals to be completely deposited. Salivary substitutes lack various enzymes, proteins, and acquired pellicle formation that dynamically alter both stain adsorption and enamel surface protection. SEM analysis was limited to two samples per group, which were evaluated qualitatively for the morphological study. Complementary quantitative assessments, such as microhardness, energy-dispersive X-ray spectroscopy elemental analysis, or surface roughness measurements, were not performed to corroborate the observed SEM surface degradation or quantify the depth of the lesion. Initial baseline variations in chromaticity (b* values) across groups may have influenced overall color shift responsiveness.

## Conclusions

WSLs treated with RI showed color change as well as enamel surface topography comparable to intact enamel post bleaching. Thus, RI can be considered a treatment modality. Bleaching done on teeth with untreated WSLs demonstrated severe enamel topographical changes, even though an appropriate color change was observed. Therefore, bleaching on teeth with enamel caries would not be recommended. Enamel caries treated with CPP-ACP depicted a smooth enamel surface even though the color change post bleaching was the least among all the groups. The efficacy of bleaching is reduced, and treatment with CPP-ACP as a remineralizing agent would need further research if bleaching is to be performed.
